# Volatile scents of influenza A and *S. pyogenes* (co-)infected cells

**DOI:** 10.1038/s41598-019-55334-0

**Published:** 2019-12-11

**Authors:** Selina Traxler, Gina Barkowsky, Radost Saß, Ann-Christin Klemenz, Nadja Patenge, Bernd Kreikemeyer, Jochen K. Schubert, Wolfram Miekisch

**Affiliations:** 1 Department of Anaesthesiology and Intensive Care, Rostock University Medical Center, Schillingallee 35, 18057 Rostock, Germany; 2 Institute of Medical Microbiology, Virology and Hygiene, Rostock University Medical Center, Schillingallee 70, 18057 Rostock, Germany

**Keywords:** Mechanisms of disease, Influenza virus, Diagnostic markers

## Abstract

Influenza A is a serious pathogen itself, but often leads to dangerous co-infections in combination with bacterial species such as *Streptococcus pyogenes*. In comparison to classical biochemical methods, analysis of volatile organic compounds (VOCs) in headspace above cultures can enable destruction free monitoring of metabolic processes *in vitro*. Thus, volatile biomarkers emitted from biological cell cultures and pathogens could serve for monitoring of infection processes *in vitro*. In this study we analysed VOCs from headspace above (co)-infected human cells by using a customized sampling system. For investigating the influenza A mono-infection and the viral-bacterial co-infection *in vitro*, we analysed VOCs from Detroit cells inoculated with influenza A virus and *S. pyogenes* by means of needle-trap micro-extraction (NTME) and gas chromatography mass spectrometry (GC-MS). Besides the determination of microbiological data such as cell count, cytokines, virus load and bacterial load, emissions from cell medium, uninfected cells and bacteria mono-infected cells were analysed. Significant differences in emitted VOC concentrations were identified between non-infected and infected cells. After inoculation with *S. pyogenes*, bacterial infection was mirrored by increased emissions of acetaldehyde and propanal. N-propyl acetate was linked to viral infection. Non-destructive monitoring of infections by means of VOC analysis may open a new window for infection research and clinical applications. VOC analysis could enable early recognition of pathogen presence and in-depth understanding of their etiopathology.

## Introduction

Influenza A is one of most frequent pathogens in the world and leads to serious disease in humans and animals. One of the main problem is, that influenza infections are often followed by secondary bacterial infections resulting in complex symptoms including pneumonia^1–5^. Typical species, playing a role in these superinfections, are gram positive bacteria such as *Streptococcus pneumoniae*, *Staphylococcus aureus*, and *Streptococcus pyogenes*^3–7^. While bacterial and viral mono-infections are well known, mechanisms and interactions during co-infection processes are not sufficiently investigated yet^6,8^. A better understanding of superinfections and diagnosis at an early stage, for choosing the right medical treatment, would be a great advantage in medicine.

Even though *S. pyogenes* plays an important role in co-infection processes^9,10^, sparse information is available and only a few articles are focusing on co-infections involving this bacteria in combination with influenza A^9–12^. *S. pyogenes* is an important pathogen with a large number of situation dependent virulence factors causing angina, toxin mediated shock syndrome, and pneumonia^13–15^. During the binding process of influenza A onto cells, sialic acids are removed through the effect of the viral proteins hemagglutinin (HA) and neuraminidase (NA)^16^. In this way, presence of influenza A virus can support bacterial adhesion because bacterial binding to cells without sialic acid is much easier^1,9^. Okamoto *et al*. showed that viral HA promoted internalization of *S. pyogenes* in epithelial cells in mice^12^.

At the moment, infection status from cells or in tissue samples can only be monitored by means of time consuming determination of cytokines or RNA^17–19^. One big disadvantage of these *in vitro* assays is the destruction of the cell culture which cannot be further used.

In recent years, trace gas analysis got more popular and important for basic research in different fields. Analysis of volatile organic compounds (VOCs), which are emitted from humans, animals, and cells, bears potential for non-invasive infection monitoring^20–24^. It is well known, that bacteria emit a broad spectrum of VOCs and studies in the past already determined VOC changes during bacterial or viral infections^25–33^. In an *in vivo* study, we recently found VOC changes in breath during influenza A infection in pigs^34^. Hence, we also expected changes of VOC profiles emitted from cells during viral infections and co-infections *in vitro*.

Since these VOCs are only present in trace concentrations, highly sensitive analytical methods are necessary. A common standardized method is VOC needle-trap micro-extraction (NTME) and compound separation and identification by means of gas chromatography coupled to mass spectrometry^35^. Analyzing VOCs during different infectious processes could provide profiles of compounds indicating viral and bacterial mono-infection and co-infections. These profiles could be compared to *in vivo* VOC profiles in order to identify potential biomarkers. This would offer a non-invasive technique for *in vitro* infection monitoring and would also raise hope for *in vivo* disease detection. Potential biomarkers could offer an alternative to common invasive examinations in health care and complement classical biochemical methods^28–30^. The aim of this study was to investigate VOC headspace profiles emitted from human cells mono- and co-infected *in vitro* by influenza A and *S. pyogenes*. The following questions were addressed in detail:

- Do VOC profiles change over time in biological cultures?
- Are there differences in VOC profiles between viral-, bacterial-, and co-infections?
- Can emitted compounds be linked to inflammation and do they mirror infection in cells?

## Results

### VOC headspace analysis

**Figure 1:**
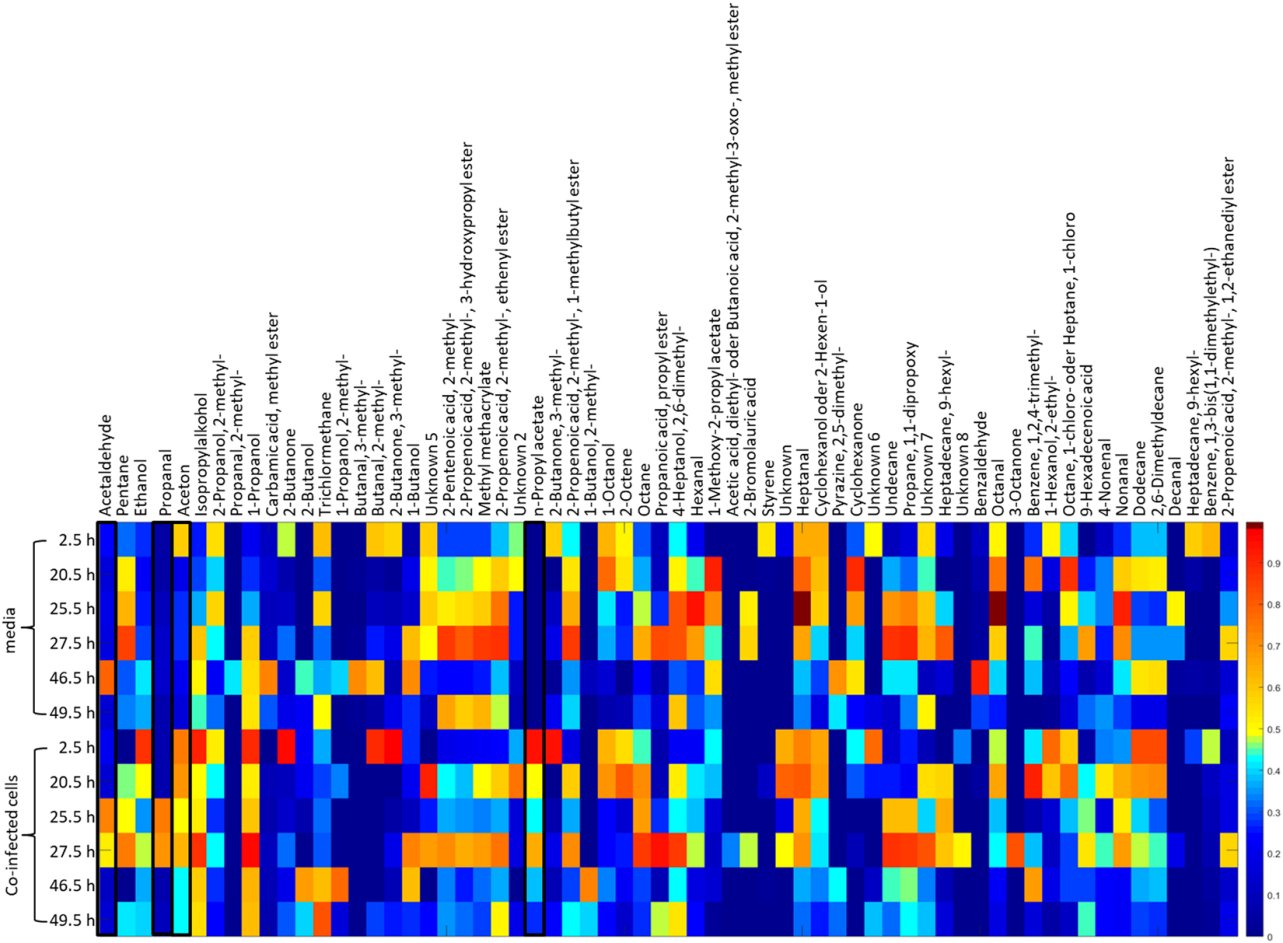
Heatmap: Target response of each compound normalized onto maximum concentration over 49.5 h.

73 substances were detected in headspaces above cultures. From these substances only 64 were consistently above LOQ (Fig. 1). Out of these, we excluded obviously exogenous compounds, such as ethanol or 2-butanone (ingredients of disinfectants). If concentration differences between control group and infected group were below 10%, substances were also sorted out. Target VOCs were only considered for detailed evaluation when concentrations above the cultures showed reproducible changes during the infection progress. Identification of the selected target compounds was then validated by means of pure reference substances, substance concentrations were determined by means of calibration in the relevant concentration range (see method section).

Acetaldehyde, propanal and n-propyl acetate showed reproducible concentration trends within infections and reproducible differences between DMEM medium, uninfected cells, influenza A infected cells, *S. pyogenes* infected cells and co-infected cells. Since these three compounds have already been identified as potential *in vivo* biomarkers during influenza A infections and acetone is a common compound in trace (breath) gas analysis, we focused on these four compounds. Limit of detection for acetaldehyde was 1.5 nmol/L, for propanal 0.12 nmol/L, for acetone 0.12 nmol/L, and for n-propyl acetate 0.0006 nmol/L. Limit of quantification (LOQ) was determined for acetaldehyde as1.8 nmol/L, of propanal as 0.15 nmol/L, for acetone as 0.17 nmol/L, and for n-propyl acetate as 0.0009 nmol/L.

**Figure 2:**
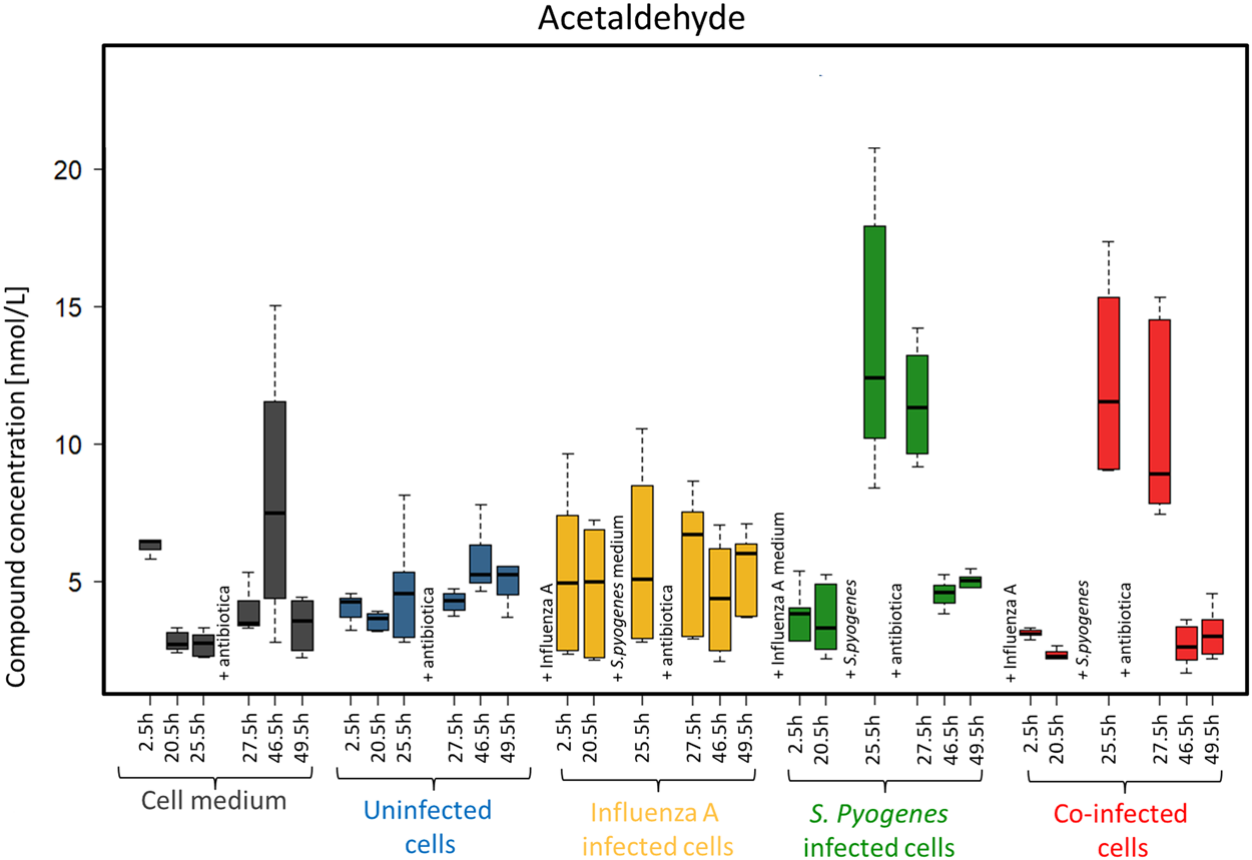
Acetaldehyde concentrations over 49.5 h emitted from media (grey), uninfected cells (blue), influenza A infected cells (yellow), *S. pyogenes* infected cells (green) and co-infected cells (red).

Acetaldehyde was emitted during all experiments (Fig. 2). Significant concentration differences are shown in Supplement Tables S3 and S4. Besides a significant increase of acetaldehyde concentrations after 25.5 hours in the pure cell medium (shown in grey), a nearly constant emission was detected from uninfected cells (shown in blue) and influenza A infected cells (shown in yellow). *S. pyogenes* infected cells (shown in green) and co-infected cells (shown in red) showed significant concentration increases after bacterial inoculation after 25.5 h and 27.5 h while concentrations were higher in *S. pyogenes* infected cells than in co-infected cells. These two concentration peaks in *S. pyogenes* infected cells and co-infected cells were significantly different from all other times of measurement within the infections and they were also significantly different from the corresponding concentrations in cell medium, uninfected cells, influenza A infected cells after 25.5 h and 27.5 h.

**Figure 3:**
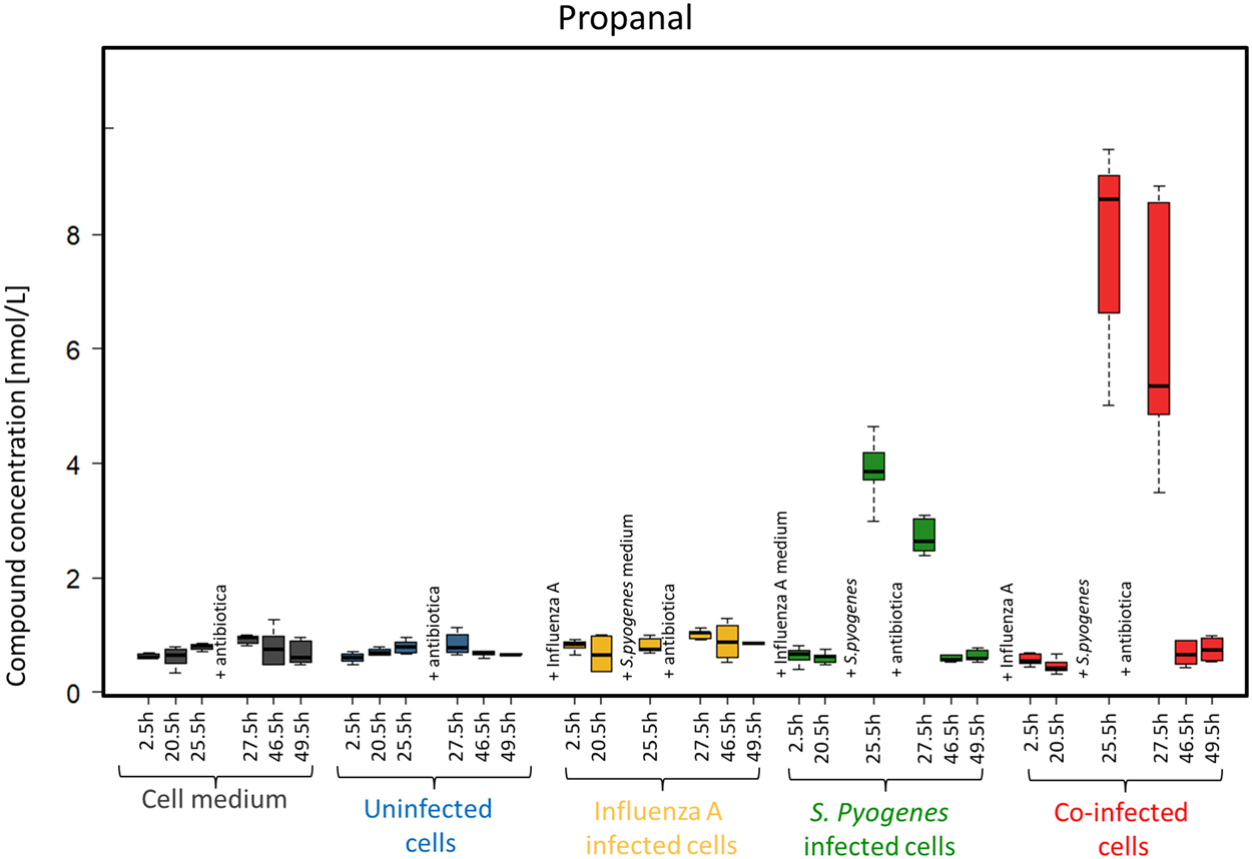
Propanal concentrations emitted over 49.5 h from media (grey), uninfected cells (blue), influenza A infected cells (yellow), *S. pyogenes* infected cells (green) and co-infected cells (red).

Propanal concentrations showed a significant increase (see Supplement Tables S3 and S4) after 25.5 h and 27.5 h in *S. pyogenes* and co-infected cells (Fig. 3).

**Figure 4:**
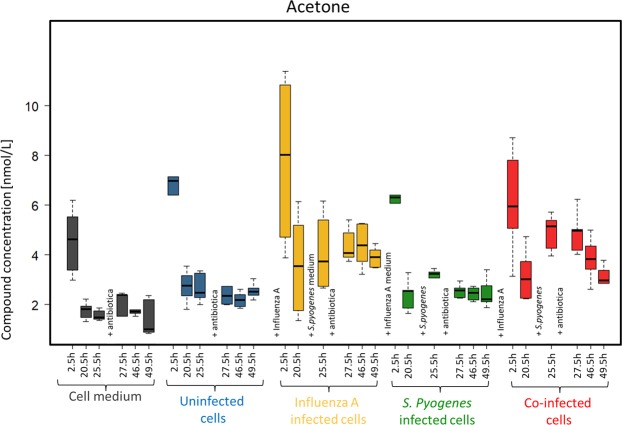
Acetone concentrations emitted over 49.5 h from media (grey), uninfected cells (blue), influenza A infected cells (yellow), *S. pyogenes* infected cells (green) and co-infected cells (red).

Acetone concentrations showed similar trends in the time course for all investigated cultures (Fig. 4) and showed no significant differences within the cultures and between the different infection setups until 25.5 h (see Supplement Table S5).

**Figure 5:**
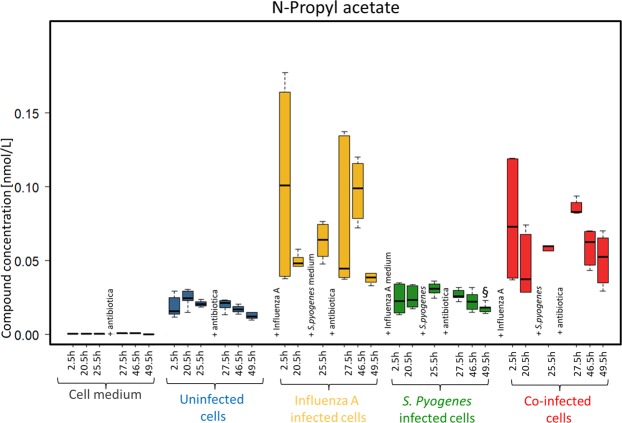
N-propyl acetate concentrations emitted over 49.5 h from media (grey), uninfected cells (blue), influenza A infected cells (yellow), *S. pyogenes* infected cells (green) and co-infected cells (red).

N-propyl acetate was detectable only in low concentrations in the headspace of cell culture media (Fig. 5). Statistical data on n-propyl acetate is shown in Supplement Table S6. While concentration ranges from uninfected cells and *S. pyogenes* infected cells were nearly constant over time, influenza A infected cells and co-infected cells showed noticeable changes during measurements. Maximum concentrations were reached in both infections after 2.5 hours. Then, n-propyl acetate decreased after 20.5 h and reached a second peak after 27.5 h and 46.5 h. All statistical values are presented in Tables S3–S6 in the Supplemental Material.

### Host cell response

#### Cell viability

At the end of VOC analyses (49.5 h), all experiments showed similar numbers in live, dead, and total cells/ml with lower values during the second *S. pyogenes* infection and higher values during the first co-infection. Data is shown in Supplement Table S2. During all experiments cell viability varied between 57.6% and 93.8% after the last VOC analysis at hour 49.5 as shown in Table S2. Cell diameter was nearly the same after all infections as shown in Supplement Table S2.

#### Secretion of Interleukin-6 and Interleukin-8

**Figure 6:**
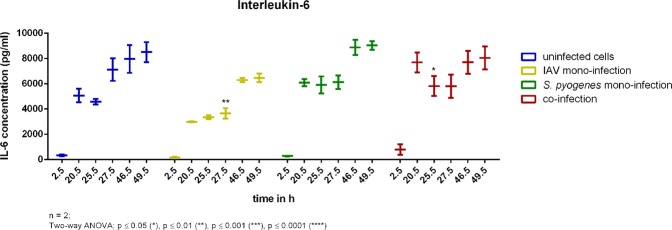
Interleukin-6 concentrations determined from uninfected cells (blue), influenza A infected cells (yellow), *S. pyogenes* infected cells (green) and co-infected cells (red) from the 24-well plate experiment over 49.5 hours. Significance was tested by means of Two-way ANOVA; *p ≤ 0.05, **p ≤ 0.01, ***p ≤ 0.001, ****p ≤ 0.0001.

Interleukin-6 concentrations in uninfected cells (shown in blue), *S. pyogenes* infected cells (shown in green), and co-infected cells (shown in red) did not show any significant differences from each other. Concentrations of Interleukin-6 was higher in the supernatant of co-infected cells (shown in red) compared to influenza A mono-infected cells (shown in yellow) after 2.5 h, 20.5 h, and 25.5 h (Fig. 6). After 27.5 h, 46.5 h and 49.5 h Interleukin-6 showed the same trend in co-infected cells as in uninfected cells.

**Figure 7:**
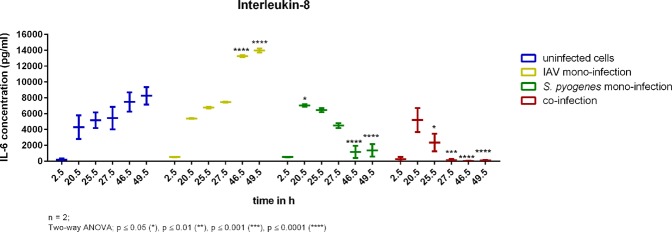
Interleukin-8 concentrations determined from uninfected cells (blue), influenza A infected cells (yellow), S. pyogenes infected cells (green) and co-infected cells (red) from the 24-well plate experiment over 49.5 hours. Significance was tested by means of Two-way ANOVA; *p ≤ 0.05, **p ≤ 0.01, ***p ≤ 0.001, ****p ≤ 0.0001.

Interleukin-8 concentrations increased in uninfected cells over the time of the experiment. Influenza A mono-infected cells showed significantly higher Interleukin-8 concentrations compared to uninfected cells, *S. pyogenes* infected cells and co-infected cells (shown in red) after 27.5 h, 46.5 h, and 49.5 h. IL-8 concentrations increased after 20.5 h in *S. pyogenes* infected cells before it decreased significantly until the end of the experiment. Co-infected cells secreted similar amounts of Interleukin-8 as uninfected cells (shown in blue) after 2.5 h and 20.5 h as shown in Fig. 7. IL-8 concentration in co-infected cells decreased in a similar way as in *S. pyogenes* infected cells after 25.5 h and 27.5 h until it was not detectable anymore after 46.5 h and 49.5 h.

#### Bacterial adherence and internalization

**Figure 8:**
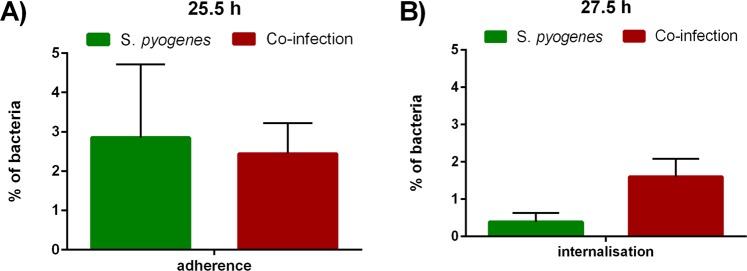
Bacterial adherence and invasion. (**A**) adherent and (**B**) internalized bacteria following *S. pyogenes* mono-infection and co-infection. Sample size n = 2.

The bacterial numbers in the supernatant reached 10^5^ CFU/ml at 1.5 h following bacterial inoculation. After adding antibiotics after 46.5 hours, no bacteria were detectable anymore. Bacteria were able to adhere to and to invade infected cells during mono-infection and co-infection (Fig. 8). 2–4% Bacteria adhered to the host cells Fig. 8(A), whereas 0.3–1.6% of *S. pyogenes* was shown to invade into infected cells Fig. 8(B). Bacterial number in the supernatant and internalized bacteria were higher during the co-infection compared to the *S. pyogenes* mono-infection.

## Discussion

*In vitro* VOC profiling during (co-)infections was possible by an adapted set-up and NTME-GC-MS analysis. We were able to identify and quantify volatile compounds over cell cultures in trace concentrations from nmol/L to pmol/L. Acetaldehyde and propanal concentrations increased significantly when *S. pyogenes* was present. In agreement with previous *in vivo* studies in pigs during an influenza A infection, n-propyl acetate was emitted in significantly higher concentrations from cells during influenza A infection^34^. Thus, n-propyl acetate is one of the first VOCs that could be identified as potential biomarker *in vivo* and could be verified on the cellular level *in vitro*. During co-infection we detected high concentrations of acetaldehyde, propanal, and n-propyl acetate.

The infection set-up was designed by combining two standardized microbiological methods of cultivation and infection for influenza A virus and *S. pyogenes*. To reduce potential confounding effects from the cell media, we analyzed effects of MEM medium addition during the *S. pyogenes* mono-infection and DMEM medium addition during the influenza A mono-infection^21,36^. Thus, we can presume that VOC changes do not occur due to medium addition but due to pathogen inoculation itself.

Although co-infection with influenza and bacteria usually takes place in the airways, we used pharynx carcinoma (Detroit) cells as infection model. These cells were chosen as they have widely been used in research of bacterial pathogenesis, especially concerning *S. pyogenes* adherence and internalization. Although the specific experiments (viral, bacterial, co-infection) were only done with two replicates, due to the experimental setup VOC analyses from the influenza A mono-infection were actually repeated four times. In combination with the observed consistent changes over time and the clear and distinct changes in VOC concentrations, results concerning influenza infection can be regarded as reproducible and robust. An additional confirmation of the validity of our data can be seen in the fact that results of the *in vitro* study are in agreement with our previous *in vivo* study in pigs during influenza A infection.

To monitor the infection status at the times of VOC analysis, co-infections were repeated in a 24-well-plate assay. Cell counting from petri dishes would only have been possible after the last VOC analysis and not during the experiment because cell counting would have affected cell cultures and VOC emissions.

Hence, cell counting was done in 24 well plates at each time of VOC analysis. Comparison of cell numbers from the petri dishes after the last VOC analysis with cell numbers in the additional 24 well plate approach showed similar values. This means, that both approaches the petri dishes and the 24 well-plates were well comparable. Since we started the experiment with already confluent Detroit cells, cell numbers did not change significantly during the experiment. Hence, correction of VOC emissions for cell growth was not done. Using our inert and standardized *in vitro* measurement system for VOC headspace sampling, we were able to obtain reproducible analyses within different infection setups. Results from the three NTDs, we sampled at each time point of VOC analysis showed (relative) variations lower than 10%. I.e. dilution effects due to consecutive sampling of three NTDs did not occur. Thus, we analyzed culture emissions produced within 1 h during each VOC analysis, with reduced surrounding confounders and without destroying the cell cultures^37^. Identification of compounds of interest was performed by validation with pure reference substances and not just by comparing the mass spectra with a database.

DMEM medium and uninfected cells showed nearly constant concentrations of relevant VOC biomarkers over the experimental time. Higher acetaldehyde emissions at the beginning of the experiment could result from the fresh preparation of the medium. Since the emissions of acetone showed the same trend in uninfected cells, infected cells and the DMEM medium, acetone emissions may be related to DMEM medium. For some other compounds, we detected considerable differences in emissions between the different infection setups. Concentration of acetaldehyde and propanal were only increasing significantly after bacterial inoculation in the *S. pyogenes* mono-culture and co-culture. Since these high emissions were not detected in DMEM medium, uninfected cells or influenza A infected cells, it seems that emissions of these two compounds depend on bacterial metabolism or host cell infection. Emissions of propanal were significantly higher during the co-infection process compared to the bacterial mono-infection. It is known, that simultaneous presence of influenza A and *S. pyogenes* can lead to relevant interactions between virus and bacteria. Removing sialic acid by viral neuraminidase for example leads to a better adherence of bacteria^16,38,39^. We detected a higher number of bacteria and more internalized bacteria during the co-infection process compared to the *S. pyogenes* mono-infection. So, the higher emissions of propanal during the co-infection process could result from increased oxidative stress in Detroit cells after increased bacterial internalization caused by the virus presence.

Interleukin-6 and -8 are general markers of inflammation and infection^40,41^. While IL-6 secretion did not change significantly following (co-)infection, interleukin 8 secretion increased significantly in influenza A mono-infected cells and, therefore, mirrors viral infection in Detroit cells. IL-8 concentrations decreased in *S. pyogenes* mono-infected and co-infected cells, most probably due to degradation of IL-8 through the cell envelope proteinase (SpyCEP), which is produced by the bacteria^42^. As bacterial load and the number of adherent bacteria increased after inoculation with *S. pyogenes* in mono-infected and co-infected cells, decreased IL-8 concentrations in the presence of *S. pyogenes* mirror bacterial infection of Detroit cells in our model. Since concentrations of acetaldehyde and propanal also increased at these times, one might assume, that emissions of acetaldehyde and propanal may mirror bacterial load and numbers of adherent bacteria. Bacterial number was well controlled in the 24 well-plates and did not correlated with VOC emissions. It is well known, that VOC emissions from cultures do often not correlate with bacterial density but rather with the growing phase of the bacteria. Previous studies already showed that lower numbers of bacteria may emit much higher concentrations of certain VOCs than a much higher number of bacteria^43^. Acetaldehyde and propanal were already detected in streptococcal species in the past and it is well known, that acetaldehyde can also be produced by *S. pyogenes* as shown in Fig. S1 ^29^. Hence, high concentrations of acetaldehyde and propanal can result from the bacteria themselves^44^. Another explanation of acetaldehyde and propanal origins could be oxidative stress induced in the cells during bacterial presence (Fig. S1). In general, infections can cause oxidative stress leading to oxidation of unsaturated fatty acids^26,45,46^. Products of this process can be acetaldehyde and propanal^47^.

As n-propyl acetate was only detectable in high concentrations during virus presence in the viral culture and co-cultures, this substance mirrors influenza A infection^1^. In the past, n-propyl acetate was detected from human fibroblasts (HFB), hepatocellular carcinoma cells (HepG2), and primary human bronchial epithelial cells (HBEpC)^48–50^. Since influenza A has not any metabolism of its own, n-propyl acetate emissions can only result from interactions between virus and cells^25^.

Influenza A expresses the surface antigen hemagglutinin, which binds to sialic acid from the cell surface. Virus is absorbed by the cell through receptor induced endocytosis. This complex process includes actions of the enzyme neuraminidase, clathrin and a lot of other proteins. Therefore, n-propyl acetate could result from this binding process or from the endocytosis. In both cases, the substance mirrors the viral infection^51,52^. Another hypothesis would be that n-propyl acetate occurs from cell metabolism induced during the infection process. Studies in the past described a modified energy production during viral infections^53^. Hence, higher concentrations of the ester n-propyl acetate could result from changes in cell metabolism as product of acetate and C3 compounds from other pathways. Emissions of n-propyl acetate showed high variations during influenza A mono-infection. Release of n-propyl acetate is most probably due to cell-virus interactions^48^, as viruses do not have their own metabolism. As emissions of n-propyl acetate from uninfected cells and *S. pyogenes* infected cells showed only minor changes, varying concentrations of n-propyl acetate mirror dynamic interactions between cells and influenza A virus rather than variations induced by methodology or analytical techniques.

Recently described *in vivo* VOC monitoring showed an increase of acetaldehyde, propanal and n-propyl acetate concentration in pigs’ breath during an influenza A infection^34^. In our *in vitro* study acetaldehyde and propanal concentration increased only in bacterial infection and co-infection and not during virus presence. Although pigs were only infected with influenza A and not with bacteria, acetaldehyde and propanal concentration increased in pigs’ breath during the viral infection, too. Since pigs have a natural bacterial population in the respiratory tract, high concentrations of acetaldehyde and propanal could result from interactions between virus and the natural microbiome in pigs during the influenza A infection. N-propyl acetate was emitted in highest concentrations in pigs’ breath during the influenza A infection. Since we already detected these high concentrations of n-propyl acetate *in vivo* and since it was emitted in significant higher concentrations in presence of influenza A *in vitro*, it seems that n-propyl acetate could serve as a potential biomarker for H1N1 infections.

To our knowledge, this is the first time that a compound identified as potential *in vivo* biomarker was also found during *in vitro* studies involving the same virus type. N-propyl acetate increased during infection *in vivo* and *in vitro* and could be used for a destruction free influenza A infection monitoring. Although emissions of acetaldehyde and propanal are not specific for *S. pyogenes* infections, the combination of all compounds is useful for recognition and monitoring of co-infections *in vitro*. From our results it is obvious that volatile emissions of cells differ, when cells are infected with viruses or bacteria. Based on these findings, non-invasive or even continuous monitoring of (co-)infection processes *in vitro* could become feasible. Such a monitoring could help to understand time course and basic mechanisms (such as cell adherence) of infections e.g. as some volatile marker substances have also been observed during viral infections *in vivo*. Further studies are necessary to check, if these results can be translated into clinical settings. Nevertheless, our study provides important and promising results for translational research in future and could help to understand course and basic mechanisms of (co)infections.

VOC analysis could enable non-invasive and destruction-free monitoring of influenza A infection status in cells *in vitro*. Characterizing a biomarker for influenza A infections would be a big step for breath gas analysis and could enable a non-invasive tool for cell culture observation and - in a perspective - foster diagnosis and monitoring of infections.

## Materials and Methods

### Cultivation of cultures

#### Eukaryotic cells

Madin-Darby canine kidney (MDCKII, Sigma-Aldrich/Merck, Darmstadt, Germany) cells were used for the cultivation of virus and virus titration. Homo sapiens pharynx carcinoma cells (Detroit 562, CLS, Eppelheim, Germany) were used for adherence assays and VOC analysis.

The MDCKII cells were maintained in MEM medium (Sigma-Aldrich/Merck, Darmstadt, Germany) with 2 mM L-glutamine and 5% fetal bovin serum (v/v, FBS, Sigma-Aldrich/Merck, Darmstadt, Germany) and the Detroit cells were cultivated in Dulbecco’s Modified Eagle’s medium (DMEM, Gibco, Thermofischer, Darmstadt, Germany) with 10% FBS.

Both cell lines were incubated at 37 °C in a 5% CO_2_ atmosphere. For the VOC analysis, two glass petri dishes with Detroit cells were prepared in DMEM.

#### Influenza A virus

Influenza A Virus A/Bayern/74/2009 (H1N1pdm09) (IAV) was kindly provided from the Federal Research Institute for animal health (FLI, Riems, Germany). MDCK II-cells were prepared in MEM-medium containing 14% bovine serum albumin (35%, MP Biomedicals, Eschwege Germany), 10000 Unit/ml penicillin/streptomycin (v/v, Gibco, Thermofischer, Darmstadt, Germany) and 2 µg/ml N-tosyl-L-phenylalanine chloromethyl ketone (TPCK)-treated trypsin (Sigma-Aldrich/Merck, Darmstadt, Germany) for the cultivation of IAV. The calculation of TCID_50_/ml was performed by following the protocol of “Virology Methods Manual, p. 374”^54^.

#### Streptococcus pyogenes

*S. pyogenes* strain AP1 was kindly provided from the World Health Organization (WHO) Collaborating Centre for Reference and Research on Streptococci (Prague, Czech Republic). Bacteria were cultured in Todd-Hewitt broth (TH, Becton Dickinson, Heidelberg, Germany) at 37 °C in the presence of 5% CO_2_. Cultures were centrifuged at 4000 g for 10 min. After washing the pellet with 5 ml 1 x phosphate buffered saline (1 x PBS) (137 mM NaCl, 2,7 mM KCl, 10 mM Na_2_HPO_4_ * 2 H_2_O, 2 mM KH_2_PO_4_), a second centrifugation at 4000 g for 10 min was done. Pellets were re-suspended in 2 ml DMEM medium and the optical density (OD_600nm_) was adjusted to 0.5. The resulting suspension was diluted 1:10.

### Experimental protocol for VOC analysis and microbiological analysis

**Figure 9:**
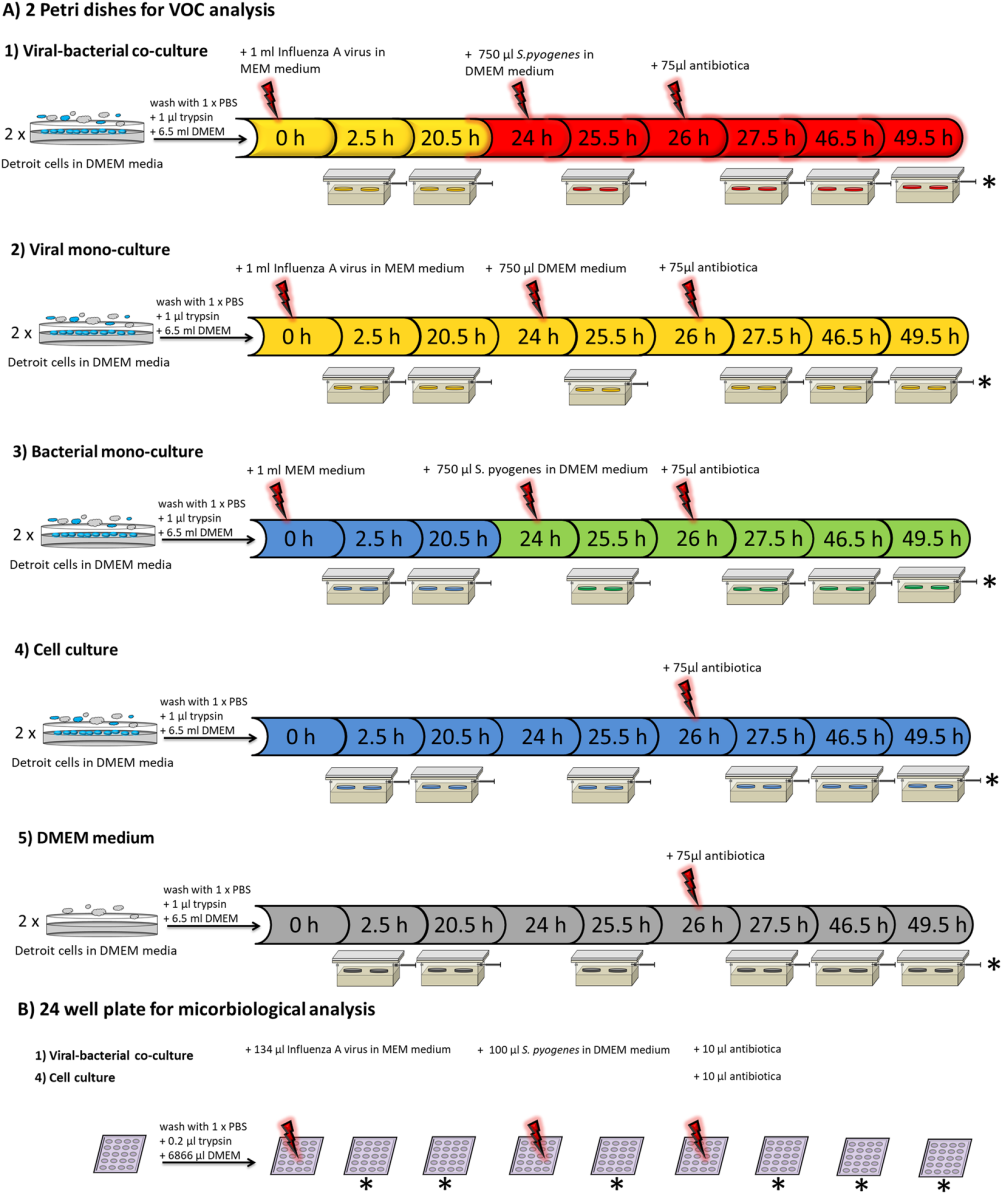
Experimental protocol for (**A**) VOC analysis and (**B**) microbiological analysis: (A-1) Two petri dishes with Detroit cells were infected with influenza A virus at hour 0 and co-infected with *S. pyogenes* after 24 hours. Times of VOC analyses are indicated by the symbol of the sampling box. After the last VOC analysis, petri dishes were used for cell counting*. The same sampling protocol was used for virus mono-infection by adding DMEM after 24 h (A-2)) and for bacterial mono-infection by adding MEM at 0 h (A-3)), and for uninfected cells and pure DMEM by adding 1% penicillin/streptomycin. Co-infection was performed in a 24 well plate for cell counting* and in parallel to VOC analyses, microbiological analyses were done.

DMEM was removed from Detroit cells and attached cells were washed with 5 ml 1 x PBS. Detroit cells were inoculated with 1 ml virus suspension (8.89 ∙ 10^5^ TCID_50_/ml). After adding 6.5 ml DMEM and 1.5 ml trypsin, the cells cultures were incubated at 37 °C with 5% CO_2_ (0 h). For VOC analysis, petri dishes were incubated for 1.5 hours and placed in the *in vitro* measurement system. First VOC samples were taken after 2.5 h and 20.5 h as shown in the experimental protocol in Fig. 9(A).

For viral-bacterial co-infection experiments, 750 µl suspension containing *S. pyogenes* were added after 24 h to the influenza mono-infection. Following another 30 minutes of incubation, the next VOC analysis was performed (25.5 h). Co-cultures were returned to the incubator for 30 minutes and treated with 1% penicillin/streptomycin (26 h). Additional VOC samples were taken after 27.5 h, 46.5 h and 49.5 h.

The same experiment was performed with viral and bacterial mono-infections by adding either DMEM to the influenza infected cells or virus medium to the cells infected with *S. pyogenes* (Fig. 9(A-2,A-3)). Cell media and cell cultures were also analysed as controls by only adding antibiotics after 26 hours (Fig. 9(A-4,A-5)). Each infection was performed in two biological replicates.

After VOC analyses were completed, petri dishes were prepared for cell counting. For determining bacterial load, bacterial adherence and interleukin concentrations at all times of VOC analyses identical experiments were performed in 24 well plates as shown in Fig. 9(B). Uninfected cells served as control.

Although the specific experiments (viral, bacterial, co-infection) were only done with two replicates, the first 3 stages of all experiments happened under identical conditions. These ten parallel assays done at (at least) three different times enabled us to assess variations induced from media, cell growth and analytical procedures.

In addition, controls consisting of pure media and cells + media were analyzed in parallel for any stage of the experiment. The first time points of influenza A mono-infection and co-infection were exactly the same, as we always inoculated cell cultures with influenza A first.

### VOC headspace analysis

#### Headspace sampling and VOC pre concentration

Needle trap devices (NTD) (Shinwa Ltd, Japan) were pre-conditioned by flushing the adsorbent-copolymer of methacrylic acid and ethylene glycol dimethacrylate with helium (Linde AG, Germany) in a heating device (PAS Technology Deutschland GmbH, Magdala, Germany) at 200 °C for 30 min as previously described^27,31,32^.

For VOC analysis two culture petri dishes were placed in the first measurement box of Teflon® and glass under the sterile hood. Another two petri dishes with DMEM medium, uninfected cell culture, or infected cells were placed in the second measurement box. Boxes were hermetically closed and connected to a vacuum pump as described before^27,31,32^. A tedlar bag (SKC Eighty Four, PA, USA) was filled with 3 l synthetic clean air containing 75% N_2_, 20% O_2_ und 5% CO_2_ (Air Liquide, Düsseldorf, Germany)^31,32^. Headspace above cultures was flushed by connecting the tedlar bag to a second adapter on the opposite side of the measurement system and using the vacuum pump. After closing the Luer-Lock adapters of the measurement system with (IN) stoppers the whole measurement system was placed in a heating chamber (Memmert GmBh + Co. KG, Schwabach, Germany) at 37 °C. After one hour, NTDs were connected to a 1 ml tuberkulin syringe (B Braun, Melsungen, Germany) and pushed through the septum of the IN stopper into the box headspace. VOC pre-concentration was done by sampling 50 ml headspace air at ambient pressure and 37 °C bi-directionally as described before^27,31,32^. From each measurement box, three replicates were taken. After sampling, NTDs were closed with Teflon caps (Shinwa Ltd, Japan) until GC-MS analysis was performed. Measurement boxes were opened under the sterile hood. Petri dishes were removed and placed back into the incubator at 37 °C. After cleaning the boxes with disinfectants, they were stored in the heating chamber at 50 °C until the next experiments.

#### VOC identification and calibration

VOC separation and identification were done as previously described^27,31,32^. An Agilent 7890 A gas chromatograph coupled to an Agilent 5975 C inert XL MSD with triple axis detector was used. VOCs were thermally desorbed from NTD and analyzed applying the parameters listed in Table 1.

**Table 1:** Parameters of analysis by means of gas chromatography coupled with mass spectrometry.

Gas chromatograph	Agilent 7890A
Desorption	thermal
Injector temperature	200 °C
Mode	splitless mode (60 s)
Column	60 m RTX- 624 column 6% (Cyanopropylphenyl/94% dimethyl polysiloxane)
Column thickness	0.32 mm ID, 1.8 μm
Carrier gas	helium
Carrier gas flow	1.5 ml/min
Temperature program	40 °C for 5 min, 8 °C/min to 120 °C for 2 min, 10 °C/min to 220 °C, 20 °C/min to 240 °C for 4.5 min
**MSD with triple axis detector**	**Agilent 5975C inert XL**
Ionization	electron impact ionization (EI −70 eV)
Scan mode SIM Ions	full scan mode with mass range 35–250 amu 41, 43, 53, 57, 62, 67, 72, 91, 106, 116, 123
Scan rate	2.73 scan/s

VOC GC-MS data was analyzed in selected ion monitoring (SIM) modus by using the MSD ChemStation (Agilent Technologies, Germany) and detected VOC mass spectra were compared to the NIST MS search data base (Version 2.0). 10 blank NTDs were analyzed for calculating limits of detection as a signal-to-noise ratio of 3:1, whereas limits of quantification corresponded to a signal-to-noise ratio of 10:1^27,31,32^. Calibration and compound verification of potential marker compounds was done by using the pure reference substances shown in Supplement Table S1.

For quantification, aqueous solutions of reference substances were evaporated by means of a liquid calibration unit (LCU, Ionicon Analytik GmbH, Innsbruck, Austria). Concentration levels of the gas standards were prepared from 1 ppb to 500 ppb by diluting the standards with nitrogen and water with a matrix adapted humidity of 25 g/m^3^ as previously described^31,32^. Evaporated standard gas was pre concentrated onto NTDs and analyzed by GC.MS.

Limits of detection (LOD) and limits of quantification (LOQ) were calculated by comparison of (measured) calibration values to the measured baseline noise. Baseline values were calculated by analyzing 10 blank NTDs after pre-conditioning. LODs and LOQs were calculated by using the triple (LOD) respectively the tenfold (LOQ) standard deviation added to the arithmetic mean of baseline noise from 10 blank NTDs.

### Analyses of cells and bacteria

#### Cell viability and cell concentration

Cell viability and cell concentration was determined using the Nucleo Counter® NC-200™ (Chemometec, Aflerod, Denmark). Cells were treated with acridine orange and 4,6-diamidino-2-phenylindole (DAPI) and analyzed by fluorescence cytometry using the “Viability and Cell Count Assay”^34^.

#### Determination of interleukins

For monitoring the infection status of the cells, interleukin-6 and -8 were determined by means of Bio-Plex Pro™ Human Cytokine Assays (Bio-Rad Laboraties GmbH, Munich, Germany) by following the instruction manual.

#### Bacterial adherence assay

At each time point of VOC analysis, bacterial adherence to Detroit cells was quantified using the antibiotic protection assay^55^. 24 well plates were inoculated with 4.5 × 10^5^ cells/ml in DMEM without antibiotics. Cells were allowed to grow until confluence. Before the assay, cells were washed twice with 1 × PBS and then incubated with virus suspension (8.89 × 10^5^ TCID_50_/ml) and 2 µg/ml trypsin within 1 ml DMEM at 37 °C. After 24 hours, 100 µl of bacterial suspension was inoculated. Two hours after bacterial infection, 1% penicillin/streptomycin was added.

At every time point of VOC analysis, supernatant was plated out on TH agar to calculate colony forming units (CFU) per ml. Detroit cells were washed with 1 × PBS, detached from the well by trypsin treatment and lysed with sterile distilled water. The CFU/ml from lysed cells was determined by serial dilution in 1 × PBS and plating on THY.

### Statistical analysis

Data handling and correlation analysis were done by means of GraphPad Prism 6. Statistical tests were performed in SigmaPlot software (version 13.0) using a one-way repeated measures analysis of variance with post-hoc “Shapiro-Wilk” and “Student-Newman-Keuls” test. Boxplots were created by means of R software (version 3.3.2) and RStudio (version 1.0.136). Concentrations of acetaldehyde and propanal determined after 20.5 h and 27.5 h were tested for significant differences between each other within the *S. pyogenes* infection and within the co-infection. Additionally, acetaldehyde and propanal concentrations in the *S. pyogenes* cultures and co-cultures were compared to the corresponding concentrations in pure media, uninfected cells and influenza infected cells. All determined n-propyl acetate concentrations were tested for significant differences between pure medium, uninfected cells, mono-infected cells, and co-infected cells.

## Supplementary information

Supplementary Information

## Data Availability

We comply with data availability policy of this journal.

## References

[1] McCullers JA (2014). The co-pathogenesis of influenza viruses with bacteria in the lung. Nat. Rev. Microbiol..

[2] Rynda-Apple A, Robinson KM, Alcorn JF (2015). Influenza and Bacterial Superinfection: Illuminating the Immunologic Mechanisms of Disease. Infect. Immun..

[3] Klein EY (2016). The frequency of influenza and bacterial coinfection: a systematic review and meta-analysis. Influenza Other Respir. Viruses.

[4] Bosch AATM, Biesbroek G, Trzcinski K, Sanders EAM, Bogaert D (2013). Viral and Bacterial Interactions in the Upper Respiratory Tract. PLOS Pathog..

[5] Chertow DS, Memoli MJ (2013). Bacterial Coinfection in Influenza: A Grand Rounds Review. JAMA.

[6] Siemens, N. *et al*. Port d’Entrée for Respiratory Infections – Does the Influenza A Virus Pave the Way for Bacteria? *Front*. *Microbiol*. **8** (2017). 10.3389/fmicb.2017.02602 PMC5742597 29312268

[7] Wang Xuan-Yi, Kilgore Paul E., Lim Kyung Ah, Wang Song-Mei, Lee Jeongseok, Deng Wei, Mo Mei-Qi, Nyambat Batmunkh, Ma Jing-Chen, Favorov Michael O., Clemens John D. (2011). Influenza and Bacterial Pathogen Coinfections in the 20th Century. Interdisciplinary Perspectives on Infectious Diseases.

[8] Hunter P (2018). Co-infection: when whole can be greater than the sum: The complex reaction to co-infection of different pathogens can generate variable symptoms. EMBO Rep..

[9] Herrera AL (2017). Binding host proteins to the M protein contributes to the mortality associated with influenza–Streptococcus pyogenes superinfections. Microbiology.

[10] Asai N (2019). A severe case of Streptococcal pyogenes empyema following influenza A infection. BMC Pulm. Med..

[11] Chaussee MS (2011). Inactivated and live, attenuated influenza vaccines protect mice against influenza:Streptococcus pyogenes super-infections. Vaccine.

[12] Okamoto S (2003). Influenza A Virus-Infected Hosts Boost an Invasive Type of Streptococcus pyogenes Infection in Mice. J. Virol..

[13] Barnham M, Weightman N, Anderson A, Pagan F, Chapman S (1999). Review of 17 cases of pneumonia caused by Streptococcus pyogenes. Eur. J. Clin. Microbiol. Infect. Dis. Off. Publ. Eur. Soc. Clin. Microbiol..

[14] Stevens DL (1989). Severe group A streptococcal infections associated with a toxic shock-like syndrome and scarlet fever toxin A. N. Engl. J. Med..

[15] Gottlieb M, Long B, Koyfman A (2018). Clinical Mimics: An Emergency Medicine-Focused Review of Streptococcal Pharyngitis Mimics. J. Emerg. Med..

[16] McCullers JA, Bartmess KC (2003). Role of Neuraminidase in Lethal Synergism between Influenza Virus and Streptococcus pneumoniae. J. Infect. Dis..

[17] De EW (2018). 1918 H1N1 Influenza Virus Replicates and Induces Proinflammatory Cytokine Responses in Extrarespiratory Tissues of Ferrets. J. Infect. Dis..

[18] Lee IH, Kim HS, Seo SH (2017). Porcine mast cells infected with H1N1 influenza virus release histamine and inflammatory cytokines and chemokines. Arch. Virol..

[19] Verma V, Kumar P, Dhanda RS, Yadav M (2016). Kinetics of cytokine profile in response to Mycobacterium bovis BCG and Streptococcus pyogenes activated cells. Data Brief.

[20] Lough F, Perry JD, Stanforth SP, Dean JR (2017). Detection of exogenous VOCs as a novel *in vitro* diagnostic technique for the detection of pathogenic bacteria. TrAC Trends Anal. Chem..

[21] van Oort Pouline M, Povoa Pedro, Schnabel Ronny, Dark Paul, Artigas Antonio, Bergmans Dennis C J J, Felton Timothy, Coelho Luis, Schultz Marcus J, Fowler Stephen J, Bos Lieuwe D (2018). The potential role of exhaled breath analysis in the diagnostic process of pneumonia—a systematic review. Journal of Breath Research.

[22] Boots AW (2014). Identification of microorganisms based on headspace analysis of volatile organic compounds by gas chromatography–mass spectrometry. J. Breath Res..

[23] Pereira J (2015). Breath Analysis as a Potential and Non-Invasive Frontier in Disease Diagnosis: An Overview. Metabolites.

[24] Sethi S, Nanda R, Chakraborty T (2013). Clinical Application of Volatile Organic Compound Analysis for Detecting Infectious Diseases. Clin. Microbiol. Rev..

[25] Aksenov AA (2014). Cellular Scent of Influenza Virus Infection. ChemBioChem.

[26] Phillips, M. *et al*. *Effect of influenza vaccination on oxidative stress products in breath*. vol. 4 (2010). 10.1088/1752-7155/4/2/026001 21383469

[27] Korpi A, Järnberg J, Pasanen A-L (2009). Microbial Volatile Organic Compounds. Crit. Rev. Toxicol..

[28] Ahmed Waqar M., Lawal Oluwasola, Nijsen Tamara M., Goodacre Royston, Fowler Stephen J. (2017). Exhaled Volatile Organic Compounds of Infection: A Systematic Review. ACS Infectious Diseases.

[29] Filipiak W (2012). Characterization of volatile metabolites taken up by or released from Streptococcus pneumoniae and Haemophilus influenzae by using GC-MS. Microbiology.

[30] Filipiak W (2012). Molecular analysis of volatile metabolites released specifically by staphylococcus aureus and pseudomonas aeruginosa. BMC Microbiol..

[31] Qader AAE (2015). Volatile organic compounds generated by cultures of bacteria and viruses associated with respiratory infections. Biomed. Chromatogr..

[32] Purcaro G (2018). Volatile fingerprinting of human respiratory viruses from cell culture. J. Breath Res..

[33] Filipiak W (2015). Breath analysis for *in vivo* detection of pathogens related to ventilator-associated pneumonia in intensive care patients: a prospective pilot study. J. Breath Res..

[34] Traxler S (2018). VOC breath profile in spontaneously breathing awake swine during Influenza A infection. Sci. Rep..

[35] Trefz P (2012). Needle trap micro-extraction for VOC analysis: Effects of packing materials and desorption parameters. J. Chromatogr. A.

[36] Bos LDJ, Sterk PJ, Schultz MJ (2013). Volatile Metabolites of Pathogens: A Systematic Review. PLOS Pathog..

[37] Traxler S, Bischoff A-C, Trefz P, Schubert JK, Miekisch W (2018). Versatile set-up for non-invasive *in vitro* analysis of headspace VOCs. J. Breath Res..

[38] Peltola VT, Murti KG, McCullers JA (2005). Influenza Virus Neuraminidase Contributes to Secondary Bacterial Pneumonia. J. Infect. Dis..

[39] Influenza Virus Neuraminidase Contributes to Secondary Bacterial Pneumonia | The Journal of Infectious Diseases | Oxford Academic, https://academic.oup.com/jid/article/192/2/249/857050 (2005). 10.1086/430954 PMC2715995 15962219

[40] Gerlach RL, Camp JV, Chu Y-K, Jonsson CB (2013). Early Host Responses of Seasonal and Pandemic Influenza A Viruses in Primary Well-Differentiated Human Lung Epithelial Cells. PLoS ONE.

[41] Kobasa D (2007). Aberrant innate immune response in lethal infection of macaques with the 1918 influenza virus. Nature.

[42] Edwards RJ (2005). Specific C-terminal cleavage and inactivation of interleukin-8 by invasive disease isolates of Streptococcus pyogenes. J. Infect. Dis..

[43] Küntzel A (2016). Effects of biological and methodological factors on volatile organic compound patterns during cultural growth of Mycobacterium avium ssp. paratuberculosis. J. Breath Res..

[44] Pancholi, V. & Caparon, M. Streptococcus pyogenes Metabolism. in *Streptococcus pyogenes: Basic Biology to* Clinical *Manifestations* (eds Ferretti, J. J., Stevens, D. L. & Fischetti, V. A.) (University of Oklahoma Health Sciences Center, 2016). 26866220

[45] Schwarz KB (1996). Oxidative stress during viral infection: A review. Free Radic. Biol. Med..

[46] Phillips M (2000). Effect of age on the breath methylated alkane contour, a display of apparent new markers of oxidative stress. J. Lab. Clin. Med..

[47] Ayala Antonio, Muñoz Mario F., Argüelles Sandro (2014). Lipid Peroxidation: Production, Metabolism, and Signaling Mechanisms of Malondialdehyde and 4-Hydroxy-2-Nonenal. Oxidative Medicine and Cellular Longevity.

[48] Filipiak W (2008). Release of volatile organic compounds (VOCs) from the lung cancer cell line CALU-1 *in vitro*. Cancer Cell Int..

[49] Mochalski P (2013). Release and uptake of volatile organic compounds by human hepatocellular carcinoma cells (HepG2) *in vitro*. Cancer Cell Int..

[50] Filipiak W (2010). TD-GC-MS Analysis of Volatile Metabolites of Human Lung Cancer and Normal Cells. In vitro. Cancer Epidemiol. Prev. Biomark..

[51] Skehel JJ, Wiley DC (2000). Receptor Binding and Membrane Fusion in Virus Entry: The Influenza Hemagglutinin. Annu. Rev. Biochem..

[52] Rust MJ, Lakadamyali M, Zhang F, Zhuang X (2004). Assembly of endocytic machinery around individual influenza viruses during viral entry. Nat. Struct. Mol. Biol..

[53] Ritter JB, Wahl AS, Freund S, Genzel Y, Reichl U (2010). Metabolic effects of influenza virus infection in cultured animal cells: Intra- and extracellular metabolite profiling. BMC Syst. Biol..

[54] Hierholzer J.C., Killington R.A. (1996). Virus isolation and quantitation. Virology Methods Manual.

[55] Ozeri V, Rosenshine I, Mosher DF, Fässler R, Hanski E (1998). Roles of integrins and fibronectin in the entry of Streptococcus pyogenes into cells via protein F1. Mol. Microbiol..

