# Supplementary material for: Volatile scents of influenza A and S. pyogenes (co-)infected cells
Source: Sci Rep. 2019 Dec 11;9:18894. doi: 10.1038/s41598-019-55334-0 (PMC6906285; doi:10.1038/s41598-019-55334-0)
Supplement: Supplementary file 1 — Supplementary Information [file 41598_2019_55334_MOESM1_ESM.docx]

# Volatile scents of influenza A and *S. pyogenes*

# (co-)infected cells

Selina Traxler^1^, Gina Barkowsky^2^, Radost Saß^1^, Ann-Christin Klemenz^1^, Nadja Patenge^2^, Bernd Kreikemeyer^2^, Jochen K Schubert^1^ and Wolfram Miekisch^1,*^

^1^ Department of Anaesthesiology and Intensive Care, Rostock University Medical Center, Schillingallee 35 18057 Rostock, Germany, wolfram.miekisch@med.uni-rosrock.de

^2^ Institute of Medical Microbiology, Virology and Hygiene, Rostock University Medical Center, Schillingallee 70 18057 Rostock, Germany, [Bernd.Kreikemeyer@med.uni-rosock.de](mailto:Bernd.Kreikemeyer@med.uni-rosock.de)

***** Correspondence: wolfram.miekisch@uni-rostock.de; Tel.: (WM) +49-381-494-5955

**Supplementary Materials:**

**Table S1.** Reference substances for VOC identification and quantification.

| **Compound** | **CAS** | **Company** |
| --- | --- | --- |
| **Acetaldehyde** | 75-07-0 | Abbot-GmbH |
| **Propanal** | 123-38-6 | Sigma Aldrich |
| **Acetone** | 67-64-1 | Sigma Aldrich |
| **n-Propyl acetate** | 109-60-4 | Sigma Aldrich |

**Table S2.** Determination of viability [%], live [cells/ml], dead [cells/ml], total [cells/ml], and cell diameter [um] from uninfected cells, influenza infected cells, *S. pyogenes* infected cells, and co-infected cells of petri dishes after last VOC analysis (49.5h)and from uninfected cells and co-infected cells of 24-well plates after 49.5 hours.

|  | **Viability [%]** | **Live [cells/ml]** | **Dead [cells/ml]** | **Total [cells/ml]** | **Cell diameter [um]** |
| --- | --- | --- | --- | --- | --- |
| **Uninfected cells 1** | 67.8 | 6.78E+05 | 3.10E+04 | 9.65E+04 | 16.8 |
| **Uninfected cells 2** | 93.8 | 6.24E+05 | 4.10E+04 | 6.65E+05 | 15.3 |
| **Influenza infected cells 1** | 67.2 | 1.81E+05 | 8.50E+04 | 2.67E+05 | 14.4 |
| **Influenza infected cells 2** | 81.4 | 5.23E+05 | 1.20E+05 | 6.42E+05 | 16.7 |
| ***S. pyogenes* infected cells 1** | 89.9 | 7.88E+05 | 8.84E+04 | 8.76E+05 | 16.5 |
| ***S. pyogenes* infected cells 2** | 57.6 | 3.65E+04 | 2.68E+04 | 6.44E+04 | 16.1 |
| **Co-infected cells 1** | 88.9 | 2.17E+06 | 2.44E+06 | 2.44E+06 | 15.3 |
| **Co-infected cells 2** | 71.45 | 1.38E+05 | 5.61E+04 | 1.94E+05 | 14.65 |
| **Uninfected cells 24-well plate 1** | 88 | n.s. | n.s. | 3.40E+05 | 14.2 |
| **Uninfected cells 24-well plate 2** | 94.1 | n.s. | n.s. | 9.50E+05 | 14 |
| **Co-infected cells 24-well plate 1** | 87.6 | n.s. | n.s. | 7.65E+05 | 14.5 |
| **Co-infected cells 24-well plate 2** | 95.6 | n.s. | n.s. | 8.50E+05 | 14.1 |

**Table S3**. P-values of significant differences within VOC analysis from acetaldehyde and propanal emitted during *S. pyogenes* and co-infected cells. Significance was tested by means of one-way repeated measures analysis of variance with post-hoc “Shapiro-Wilk” and “Student-Newman-Keuls” test.

| **VOC analysis** | **Acetaldehyde** | | **Propanal** | |  |
| --- | --- | --- | --- | --- | --- |
|  | **P *S. pyogenes* infected cells** | **P co-infected cells** | **P *S. pyogenes* infected cells** | **P co-infected cells** | |
| **25.5h vs. 2.5h** | <0.001 | <0.001 | <0.001 | <0.001 | |
| **25.5h vs. 20.5h** | <0.001 | <0.001 | <0.001 | <0.001 | |
| **25.5h vs. 46.5h** | <0.001 | <0.001 | <0.001 | <0.001 | |
| **25.5h vs. 49.5h** | <0.001 | <0.001 | <0.001 | <0.001 | |
| **27.5h vs. 2.5h** | <0.001 | <0.001 | <0.001 | <0.001 | |
| **27.5h vs. 20.5h** | <0.001 | <0.001 | <0.001 | <0.001 | |
| **27.5h vs. 46.5h** | <0.001 | <0.001 | <0.001 | <0.001 | |
| **27.5h vs. 49.5h** | <0.001 | <0.001 | <0.001 | <0.001 | |
| **25.5h vs. 27.5h** | - | - | <0.001 | - | |

**Table S4.** P-values of significant differences from Acetaldehyde and propanal between *S. pyogenes* infected cells and co-infected cells compared to media, uninfected cells, and influenza A infected cells after 25.5h and 27.5h. Significance was tested by means of one-way repeated measures analysis of variance with post-hoc “Shapiro-Wilk” and “Student-Newman-Keuls” test.

| **Comparison** | **P Acetaldehyde** | | **P Propanal** | |
| --- | --- | --- | --- | --- |
|  | **25.5h** | **27.5h** | **25.5h** | **27.5h** |
| ***S. pyogenes* infected cells vs. Medium** | <0.001 | <0.001 | <0.001 | 0.044 |
| ***S. pyogenes* infected vs. uninfected cells** | 0.003 | <0.001 | <0.001 | 0.034 |
| ***S. pyogenes* vs. influenza infected cells** | 0.008 | 0.009 | <0.001 | - |
| **Co-infected cells vs. medium** | 0.003 | 0.004 | <0.001 | <0.001 |
| **Co-infected vs. uninfected cells** | 0.014 | 0.004 | <0.001 | <0.001 |
| **Co-infected vs. influenza infected cells** | 0.037 | 0.035 | <0.001 | <0.001 |
| **Co-infected vs. *S. pyogenes* infected cells** | <0.001 | - | - | <0.001 |

**Table S5.** P-values significant differences in emissions of Acetone between media, uninfected cells, influenza A infected cells, S-pyogenes infected cells and co-infected cells on each time point of VOC analysis. Significance was tested by means of one-way repeated measures analysis of variance with post-hoc “Shapiro-Wilk” and “Student-Newman-Keuls” test.

| **Acetone** | **P 2.5h** | **P 20.5 h** | **P 25.5h** | **P 27.5h** | **P 46.5h** | **P49.5h** |
| --- | --- | --- | --- | --- | --- | --- |
| **Uninfected cells vs. medium** |  |  |  |  |  |  |
| **Influenza infected cells vs. medium** |  |  | <0.001 | <0.001 | 0.001 | <0.001 |
| **Influenza vs. uninfected cells** |  |  |  |  |  | 0.013 |
| **Influenza vs. *S. pyogenes* infected cells** |  |  |  | <0.001 |  | 0.014 |
| **Influenza vs. co-infected cells** |  |  |  |  |  |  |
| ***S. pyogenes* infected cells vs. medium** |  |  | 0.038 |  |  |  |
| ***S. pyogenes* vs. uninfected cells** |  |  |  |  |  |  |
| **Co-infected cells vs. medium** |  |  | <0.001 | <0.001 | 0.001 | 0.006 |
| **Co-infected vs. uninfected cells** |  |  | 0.003 | <0.001 |  |  |
| **Co-infected vs. *S. pyogenes* infected cells** |  |  | 0.021 | <0.001 |  |  |

**Table S6.** P-values significant differences in emissions of n-Propyl acetate between media, uninfected cells, influenza A infected cells, S-pyogenes infected cells and co-infected cells on each time point of VOC analysis. Significance was tested by means of one-way repeated measures analysis of variance with post-hoc “Shapiro-Wilk” and “Student-Newman-Keuls” test.

| **n-Propyl acetate** | **P 2.5h** | **P 20.5 h** | **P 25.5h** | **P 27.5h** | **P 46.5h** | **P49.5h** |
| --- | --- | --- | --- | --- | --- | --- |
| **Uninfected cells vs. medium** | 0.005 | - | <0.001 | - | 0.040 | - |
| **Influenza infected cells vs. medium** | 0.009 | <0.001 | <0.001 | <0.001 | <0.001 | <0.001 |
| **Influenza vs. uninfected cells** | 0.024 | 0.012 | <0.001 | 0.004 | <0.001 | <0.001 |
| **Influenza vs. *S. pyogenes* infected cells** | 0.034 | 0.026 | <0.001 | 0.008 | <0.001 | <0.001 |
| **Influenza vs. co-infected cells** | - | - | - | - | <0.001 |  |
| ***S. pyogenes* infected cells vs. medium** | 0.003 | - | <0.001 | - | 0.009 | 0.018 |
| ***S. pyogenes* vs. uninfected cells** | - | - | 0.047 | - | - | - |
| **Co-infected cells vs. medium** | <0.001 | - | <0.001 | <0.001 | <0.001 | <0.001 |
| **Co-infected vs. uninfected cells** | 0.011 | - | <0.001 | 0.001 | <0.001 | <0.001 |
| **Co-infected vs. *S. pyogenes* infected cells** | 0.029 | - | <0.001 | 0.002 | <0.001 | <0.001 |


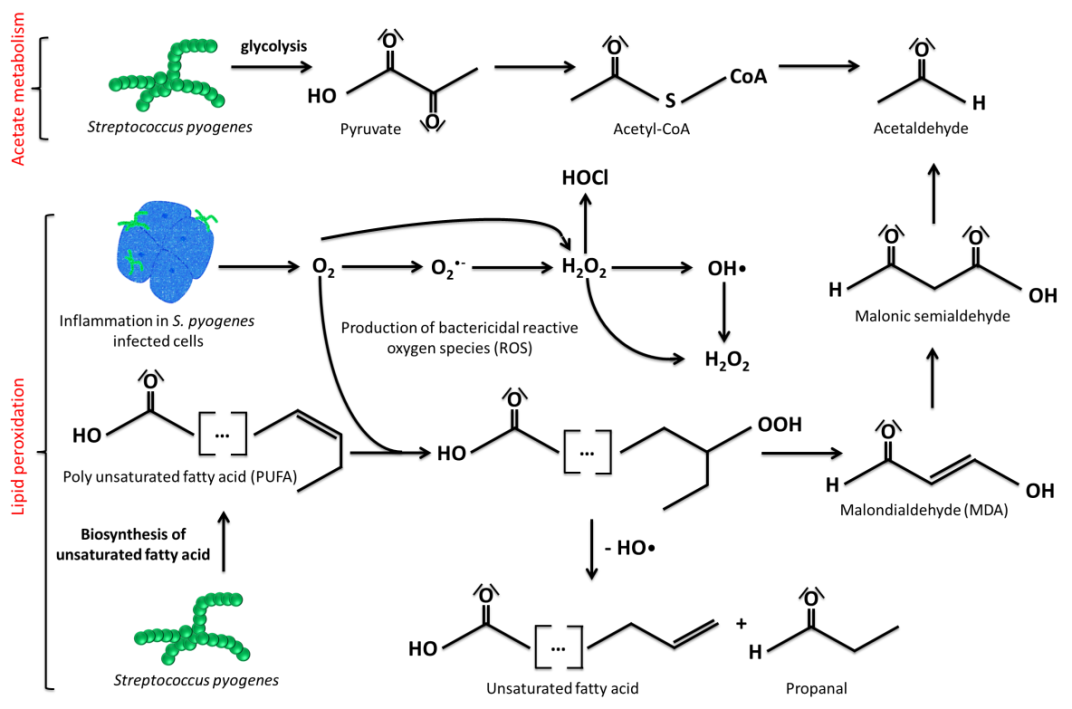


Figure S1. Possible origin of acetaldehyde and propanal during *S. pyogenes* presence from bacterial metabolism or from lipid peroxidation caused from oxidative stress during bacterial infection. [45]^,^ [48]
